# Stealth Omicron: A Novel SARS-CoV-2 Variant That Is Insensitive to RT-qPCR Using the N1 and N2 Primer-Probes

**DOI:** 10.7759/cureus.36373

**Published:** 2023-03-19

**Authors:** Hideo Mori, Hiroko Yoshida, Hideharu Mori, Tomoya Shiraki, Koichi Kawakami

**Affiliations:** 1 Pathology, Osaka Habikino Medical Center, Osaka, JPN; 2 Clinical Diagnosis, Osaka Habikino Medical Center, Osaka, JPN; 3 Laboratory of Molecular and Developmental Biology, National Institute of Genetics, Mishima, JPN

**Keywords:** mutation, s gene, n gene, omicron, rt-qpcr, sars-cov-2

## Abstract

Severe acute respiratory syndrome coronavirus 2 (SARS-CoV-2) is a novel coronavirus that is causing a worldwide pandemic since the spring of 2020. In Osaka, the second biggest prefecture in Japan, we identified a novel SARS-CoV-2 variant from a coronavirus disease 2019 (COVID-19) patient that was detected by polymerase chain reaction (PCR) using E primers, but not by real-time quantitative reverse transcription polymerase chain reaction (RT-qPCR) using the N1 and N2 primer-probe sets recommended by CDC. We analyzed the S and N gene regions by reverse-transcription and nested PCR using the S and N specific primers, and DNA sequencing, and found that this BA.5 variant contained point mutations in the probe sequences of both the N1 and N2 primer-probe regions. This finding led us to affirm the importance of monitoring the genome sequence of the SARS-CoV-2 variants continuously.

## Introduction

Coronavirus disease 2019 (COVID-19) was first noted in November 2019 in Wuhan, China, and WHO declared a global pandemic on March 11, 2020. Severe acute respiratory syndrome coronavirus 2 (SARS-CoV-2) is the virus responsible for causing the serious life-threatening COVID-19, and its genome sequence had been analyzed and determined [1,2]. CDC provided the sequences of primer-probe sets for real-time quantitative reverse transcription polymerase chain reaction (RT-qPCR), which is a gold standard test to detect the existence of SARS-CoV-2 and diagnose the infection [3]. According to this, primer-probe sets that were set in two regions in the N gene, named N1 and N2, have been adopted to RT-qPCR kits, such as the SARS-CoV-2 direct detection RT-qPCR kit (Shiga, Japan: Takara Bio Inc.). Since the pandemic began, a number of variants containing mutations in many places of the SARS-CoV-2 genome have emerged, and, at present, the Omicron variants are dominant in the world and also in Japan [4]. Rapid antigen tests (RATs) are the other important tests for the diagnosis of infection because of their speed, although their sensitivities are much less in comparison to the RT-qPCR test. Recently, it has been shown that the sensitivities of RATs, which are designed to detect the early-pandemic SARS-CoV-2 variant, are further decreased against Omicron variants, presumably due to mutations in the N protein [5]. In the present study, we report a novel variant that is insensitive to a standard RT-qPCR test using N1 and N2 primer-probes.

## Case presentation

In mid-October 2022 in Osaka, the third biggest prefecture in Japan, we examined a patient, a female in her 40s, who suffered from fever and a sore throat. We obtained the sputum sample from the patient and analyzed it by Xpert Xpress SARS-CoV-2 (Sunnyvale, CA: Cepheid) that uses primer-probe sets in the E gene (the primer sequence was not disclosed by the company) and N gene (N2). RT-qPCR for E primers showed Ct-value of 29.1, but, unexpectedly, RT-qPCR for N2 did not show positive result. Then we examined the sample by SARS-CoV-2 direct detection RT-qPCR kit (Shiga, Japan: Takara Bio Inc.) that uses the N1 and N2 primer-probe sets recommended by CDC, but could not get a positive result. Next day, we re-examined the same sample by using TRCReady SARS-CoV-2 (Tokyo, Japan: Tosoh Bioscience) and got a positive result. However, we observed some abnormal changes in the amplification curve, namely showing a slower rising pattern than usual. The patient’s symptoms of fatigue, low-grade fever, sore throat, and cough lasted three days and were relieved without sequelae. Although we could diagnose the patient as COVID-19 positive, since we identified such abnormal findings in the sputum sample, we decided to further analyze the sample more in detail.

**Figure 1:**
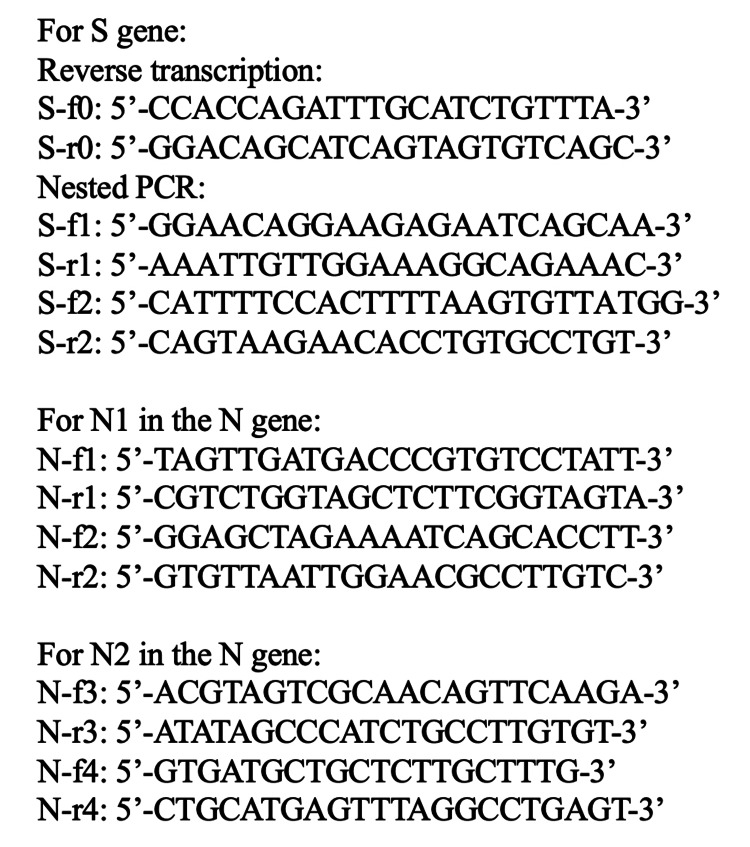
Primers for reverse transcription, nested PCR, and DNA sequencing. RNA was prepared from the sputum sample with QIAamp viral RNA mini kit (Hilden, Germany: QIAGEN). Then the viral RNA was used for reverse transcription by using PrimeScript 1st strand cDNA synthesis Kit (Shiga, Japan: Takara Bio Inc.) and primers S-f0/S-r0, N-f1/N-r1, and N-f3/N-r3. The synthesized cDNA was used for nested PCR by using Takara Ex-Taq (Shiga, Japan: Takara Bio Inc.) and S-f1/S-r1 primers followed by S-f2/S-r2 primers, N-f1/N-r1 followed by N-f2/N-r2, and N-f3/N-r3 followed by N-f4/N-r4. The synthesis of the PCR products was checked by gel electrophoresis and used for Sanger sequencing at Europhins Genomics (Japan). PCR: polymerase chain reaction

First, we analyzed the S gene region of the same sputum sample from the patient (Figure 1). We prepared RNA from the sample, synthesized cDNA by reverse transcription (RT) using the S gene-specific primers S-f0/S-r0, that covered the RBD domain, and then amplified the cDNA by nested PCR using S-f1/S-r1 followed by S-f2/S-r2. Although amplification by RT-qPCR using the N1 and N2 primer-probe sets was unsuccessful, RT and nested PCR using the S gene-specific primers could generate a DNA band. We sequenced the region 22705-23200 of the S gene and identified G22775A, A22786C, G22813T, T22917G, G22992A, C22995A, A23013C, T23018G, A23055G, A23063T, and T23075C mutations, which are commonly found in the BA.5.2 variant.

**Figure 2:**
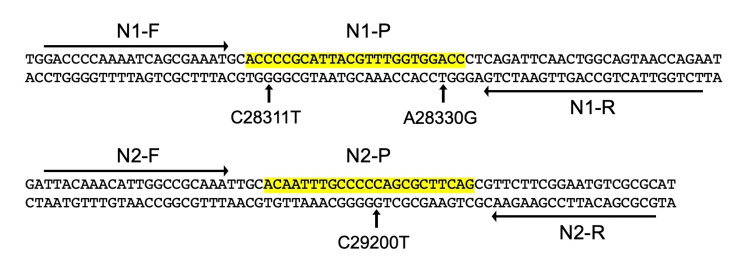
N1 and N2 primer-probe sets in the N gene and mutations. N1 and N2 primer-probe sets were recommended by CDC [3]. Arrows (N1-F, N1-R, N2-F, and N2-R) show the positions of the primers. Yellow shadows indicate the positions of the TaqMan probes (N1-P and N2-P). C28311T, A28330G, and C29200T mutations were found in the patient’s sample. We used NC_045512 (GenBank) as a reference genome of SARS-CoV-2 and the mutations identified by sequencing were analyzed by covSPECTRUM (https://cov-spectrum.org/).

Then we hypothesized the presence of unknown mutations in the N gene and designed two primer sets for reverse transcription and nested PCR that cover the N1 and N2 regions, respectively (Figure 1). RT and nested PCR using these N1 and N2-specific primers could amplify these two regions, and we sequenced the PCR products. We obtained sequences of the regions 28206-28449 for N1 and 28971-29521 for N2, and identified A28271T, C28311T, A28330G, del(28362-28370), A28402G, C29200T, C29272T, and A29510C mutations. C28311T and A28330G were located in the probe sequence of the N1 primer-probe set, and C29200T was located in the probe sequence of the N2 primer-probe set. These should have abolished detection by the RT-qPCR kit using the N1 and N2 primer-probe sets (Figure 2).

**Figure 3:**
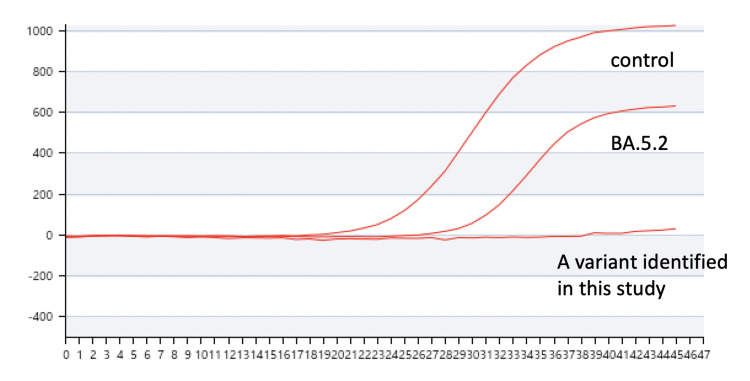
Amplification curves of Omicron variants by RT-qPCR using N1 and N2 primer-probe sets. Control RNA, BA.5.2 carrying two mutations in the N1 probe, and a variant identified in this study carrying two mutations in the N1 probe and one mutation in the N2 probe were analyzed by RT-qPCR using SARS-CoV-2 direct detection RT-qPCR kit (Shiga, Japan: Takara Bio Inc.). Vertical axis: fluorescence. Horizontal axis: PCR cycles. RT-qPCR: real-time quantitative reverse transcription polymerase chain reaction; PCR: polymerase chain reaction

We analyzed a BA.5.2 sample from another patient diagnosed around the same time in Japan carrying C28311T and A28330G (in N1), but not C29200T (in N2), by SARS-CoV-2 direct detection RT-qPCR kit (Shiga, Japan: Takara Bio Inc.). We confirmed that it could show a positive result, but showed an abnormal amplification curve presumably because the N1 probe set could not work while the N2 probe could still work (Figure 3).

## Discussion

We analyzed a SARS-CoV-2 sample found in Osaka, Japan, which could not be detected by using RT-qPCR kits using the N1 and N2 primer-probe sets, and identified mutations, G22775A, A22786C, G22813T, T22917G, G22992A, C22995A, A23013C, T23018G, A23055G, A23063T, and T23075C in the S gene and mutations A28271T, C28311T, A28330G, del(28362-28370), A28402G, C29200T, C29272T, and A29510C in the N gene. Variants carrying all of these mutations except C29200T and C29272T are mostly designated as BF.5 (covSPECTRUM: https://cov-spectrum.org/). BF.5 variants carrying C29272T are rare in Japan, and any BF.5 variants carrying both C29200T and C29272T have been identified neither in Japan nor in the world (as of March 1, 2023, covSPECTRUM: https://cov-spectrum.org/). We think that this novel “stealth” Omicron variant, carrying triple mutations in the N1 and N2 regions, could have been generated sporadically from BF.5 in Osaka, Japan.

Also, our present study demonstrated that SARS-CoV-2 variants carrying C28311T, A28330G, and C29200T may not have been detected by RT-qPCR using the N1 and N2 primer-probe sets. SARS-CoV-2 variants carrying C28311T, A28330G, and C29200T have been identified with incidences of 0.23% (80% of them were designated as BF.5) and 0.06% of the total sequences in Japan and in the world, respectively (as of March 1, 2023, covSPECTRUM: https://cov-spectrum.org/). These incidences may be underestimates since the genome analysis should have been performed by using PCR-positive samples. Thus, our present study points out a risk that some COVID-19 patients may be overlooked if they are examined only by an RT-qPCR system using the N1 and N2 primer-probe sets.

## Conclusions

At present, we do not know how broadly the “stealth Omicron” identified in this study is spreading in Japan. Based on the current finding, we propose that it is important to keep monitoring the variant at molecular levels, namely PCR and genome sequencing, and the diagnostic kit should be continuously updated as new variants emerge. Unlike RATs, which require more time-consuming efforts to be adapted to new mutations, RT-qPCR tests will easily be adapted to them by designing new primer-probe sets. Also, we recommend the PCR test should be redundant, namely examining other domains at the same time to avoid leaving patients undiagnosed.
